# Awareness and Working Intentions Toward Home-Based Care Among Macau Nurses

**DOI:** 10.1097/NHH.0000000000001373

**Published:** 2025-09-08

**Authors:** Ion Hong Wong, Kumsun Lee

**Affiliations:** **Ion Hong Wong**, is a Lecturer, Kiang Wu Nursing College of Macau.; **Kumsun Lee**, is a Professor, Faculty of Nursing, Kansai Medical University.

Macau is a rapidly aging country, with over 14% of its population aged 65 or above, and is projected to become a hyper-aging society by 2036 (Statistics and Census Service, 2014, 2022). As the world's second most densely populated region, Macau faces severe constraints in expanding institutional long-term care services—nursing home bed capacity is limited, and average waiting time for admission exceeds 1.5 years (Macau Daily, 2022). Community-based care, especially home-based services, is a viable alternative. However, only 16% of nurses currently work in non-hospital settings, and awareness for home-based care remains low (Statistics and Census Service, 2023). Although studies have examined retention in home-based care, few have explored the intention to enter this field (Park & Oh, 2014; Tourangeau et al., 2014; Yoshimura et al., 2004). This study aims to examine the awareness and working intentions of nurses in Macau toward home-based care.

## Methods

A cross-sectional survey was conducted using a structured, anonymous questionnaire. Participants were recruited through a nursing association and represented various clinical settings. Eligible participants were registered nurses capable of reading Chinese and working in hospitals, health centers, or long-term care facilities. The questionnaire included sections on demographics, attitudes toward home-based care, awareness of home-based care for older adults, and filial responsibility. One item measuring intention (“At some point I will become a home care nurse”) was used as the binary outcome in logistic regression.

## Results

Of 266 valid responses, 62.3% of nurses expressed interest in home-based care, yet only 38.5% were willing to consider it as a career (Table 1). Awareness of specific home-based care characteristics was limited, particularly in distinguishing it from institutional care and understanding available social resources. Nurses in private hospitals and long-term care facilities were significantly more likely to express intention to work in home-based care than those in government hospitals.

**Table 1.** Participants Demographics

	*N* = 236	
	*N*	%
Age (years)		
20-29	114	48.3
30-39	78	33.1
40-49	26	11.0
50-59	18	7.6
Gender		
Male	38	16.1
Female	198	83.9
Household structure		
Single household	22	9.3
Nuclear family	182	77.1
Three generations	32	13.6
Educational level		
Diploma	16	6.8
Bachelor's degree	202	85.6
Master or higher	18	7.6
Experience as a nurse		
<3 years	71	30.1
3-7 years	52	22.0
7-10 years	40	16.9
>10 years	73	30.9
Place of employment at present		
Government hospital	109	46.2
Health care center	26	11.0
Private hospital	23	9.7
Long-term care facilities	78	33.1
Position		
Nurse	211	89.4
Certificate nurse	9	3.7
Head nurse or deputy head nurse	12	5.1
Manager or deputy manager	4	1.7
Working experience in long-term care facilities		
Yes	78	33.1
No	158	66.9
Experience in home-based nursing		
Yes	84	35.6
No	152	64.4
Experience in discharge support for older people		
Yes	120	50.8
No	116	49.2
Awareness of home-based nursing		
I know well	34	14.4
I have heard about it	183	77.5
I don't know at all	19	8.1
Awareness of home-based care services in Macau		
I know well	29	12.3
I know a little	159	67.4
I don't know very well	36	15.3
I don't know at all	12	5.1
Interested in home-based care for older people		
Yes	147	62.3
No	89	37.7

Regression analysis showed that nurses with greater awareness of home-based care and those expressing interest in home-based care for older adults were significantly more likely to consider home-based care as a career (odds ratios >6.0, *p* < .01). In contrast, higher filial responsibility scores were associated with decreased intention, suggesting possible economic or cultural burdens influencing decision-making.

## Discussion

Despite high interest, a substantial gap exists between nurses' attitudes and their actual intention to work in home-based care. This may stem from insufficient exposure to the unique demands and support systems required in this field. Educational opportunities and practical training in home-based care are limited in both academic and hospital settings in Macau. Nurses who lack this experience may perceive home care as uncertain or less attractive, especially given the lower compensation packages compared to hospital-based roles. Furthermore, cultural expectations around filial duty may discourage nurses from taking lower-paying community roles, particularly when they are also caregivers for elderly family members. This highlights the need for structural reforms both in compensation and in training access to bridge the intention-action gap. Integrating home care services within hospital systems can create clearer career pathways and interdisciplinary collaboration, facilitating smoother discharge processes and continuity of care. Enhancing visibility and institutional support for home-based roles may shift perceptions and attract more nurses to this field.

## Conclusion

This study found that awareness of home-based nursing, interest in home-based care for older people, and attitudes toward filial responsibility significantly influence the intention to work in home-based care. Systematically integrating home-based care education into the curriculum, expanding clinical study opportunities in home-based care, and organizing home-based courses for working nurses can enhance awareness and interest, thereby increasing their intention to work in home care. Additionally, improving remuneration and welfare structures for nurses can reduce disparities between private and public healthcare institutions. Further research is needed to identify factors affecting the retention or resignation of home-based care nurses.

## Social Robots Help Out (https://newsinhealth.nih.gov/2025/06/medical-robots-rescue)

Zhao and others are studying how human-like, interactive robots could help people with dementia and their caregivers. As the U.S. population ages, there's a growing need for caregivers. Family caregivers often provide countless hours of support. That can lead to a lot of stress.

Zhao and his team interviewed patients and caregivers at assisted living facilities, senior centers, and memory clinics. They asked about their needs and challenges. They then customized a four-foot tall, human-like robot. It has a touch screen on its chest. These AI-powered robots can listen, talk, move, play videos, and dance. They can also encourage people to reminisce about earlier experiences.

“Patients with Alzheimer's can have short-term memory loss,” Zhao says. “But they may be able to recall what happened 20 or 30 years ago.”

The robots can play old songs, discuss sports, or ask questions to trigger memories.

“Reminiscing may not restore memory, but it can improve emotional well-being and quality of life,” Zhao says. Robots can patiently listen to the same stories over and over.

Zhao's group is also developing robots that can assist caregivers by providing evidence-based information about dementia. The robots can give tips to help caregivers reduce their own stress and stay healthy.

## References

[R1] Macau Daily. (2022, April 22). *The average waiting time for elderly people to enter residential care is half a year*. http://www.macaodaily.com/html/2022-04/27/content_1593154.htm.

[R2] Park J. Oh Y. (2014). Factors influencing turnover intention of customized home health care nurse. *Journal of Agricultural Medicine and Community Health*, 39(2), 94–103.

[R3] Statistics and Census Service, Macau Government; DESC. (2014). *The trend and challenges of population aging*. https://www.dsec.gov.mo/getAttachment/f5ecdbb4-ad4e-47fb-9937-98850909b844/pdf1.aspx?disposition=attachment.

[R4] Statistics and Census Service, Macau Government; DESC. (2022). *2021 Census Detailed Results (Updated Edition)*. https://www.dsec.gov.mo/getAttachment/b9cf8539-4731-48a9-8319-116d761ea03a/C_CEN_PUB_2021_Y.aspx.

[R5] Statistics and Census Service, Macau Government; DESC. (2023). *2022 Health Statistics*. https://www.dsec.gov.mo/getAttachment/422e039f-cd98-4ca4-b856-13546ac08b0e/C_SAU_PUB_2022_Y.aspx.

[R6] Tourangeau A. Patterson E. Rowe A. Saari M. Thomson H. MacDonald G. Cranley L. Squires M. (2014). Factors influencing home care nurse intention to remain employed. *Journal of Nursing Management*, 22(8), 1015–1026. 23905629 10.1111/jonm.12104

[R7] Yoshimura E. Yamada K. Sasagi M. (2004). Analysis of employment promotion factors of home care among mid-level nurses. *Journal of the Japan Academy of Nursing Science*, 35:82–84.

